# Supplementary material for: Biological factors associated with long COVID and comparative analysis of SARS-CoV-2 spike protein variants: a retrospective study in Thailand
Source: PeerJ. 2024 Aug 19;12:e17898. doi: 10.7717/peerj.17898 (PMC11340629; doi:10.7717/peerj.17898)
Supplement: Supplemental Information 3 [file peerj-12-17898-s003.docx]

STROBE Statement—Checklist of items that should be included in reports of ***restrospective*** ***cross-sectional studies***

|  | Item No | Recommendation | Page No. | Relevant text from manuscript |
| --- | --- | --- | --- | --- |
| **Title and abstract** | 1 | (*a*) Indicate the study’s design with a commonly used term in the title or the abstract | Title/1 | Biological factors associated with long COVID and comparative analysis of SARS-CoV-2 spike protein variants: a retrospective study in Thailand |
|  |  | (*b*) Provide in the abstract an informative and balanced summary of what was done and what was found | Abstract/2 | **Methods.** A retrospective cross-sectional study was established to recruit confirmed COVID-19 participants at Maharat Nakhon Ratchasima Hospital who had recovered for at least three months and were infected between June 2021 and August 2022. The demographic data and long COVID experience were collected via telephone interview. The biological factors were analyzed through binary logistic regression. **Results.** Data was collected from a total of 247 participants comprising 106 and 141 participants of the Delta and Omicron epidemic periods, respectively. Apart from the COVID-19 severity and health status, the baseline participant data of the two time periods were remarkably similar. The prevalence of long COVID observed in the Omicron period was higher than in the Delta period (74.5% vs. 66.0%). The biological factors associated with long COVID were epidemic variant, age, treatment with symptomatic medicines, and vaccination status. |
| Introduction | | |  |  |
| Background/rationale | 2 | Explain the scientific background and rationale for the investigation being reported | 3 | Since the beginning of COVID-19 era until the present, Thailand has been encountered with three unique epidemic SARS-CoV-2 variants: Alpha (April to early June 2021), Delta (June to November 2021), and Omicron (December 2021 until now). The Department of Disease Control of the Ministry of Public Health in Thailand has documented a cumulative count of 4,758,116 confirmed cases and 34,547 fatalities attributed to COVID-19 as of January 28, 2024 resulting in an accumulated case fatality rate of 0.73% (*DDC, 2024*). Although recovery and a low mortality rate of 3–5% have been observed in the majority of individuals infected with COVID-19, a considerable proportion of patients have experienced long-term complications referred to as long COVID (*Batiha et al., 2022*).  Besides the genetic variants of SARS-CoV-2, long COVID might be connected to several biological host variables. The development of long COVID has been associated with several characteristics, including the severity of the illness, aging, gender, and rising levels of certain inflammatory markers (Batiha et al., 2022). Nevertheless, the data pertaining to the Asian population, particularly Thai, is extremely limited. |
| Objectives | 3 | State specific objectives, including any prespecified hypotheses | 4 | This study aimed to identify biological risk factors associated with the development of long COVID in Thai, specifically focusing on the SARS-CoV-2 variants and host factors. |
| Methods | | |  |  |
| Study design | 4 | Present key elements of study design early in the paper | 5 | **Study design and participants**  This cross-sectional study collected retrospective data from confirmed COVID-19 cases who acquired the SARS-CoV-2 infection during the Delta and Omicron epidemic periods, as reported by the Department of Medical Science of the Ministry of Public Health in Thailand (DMSc, 2023). Data were analyzed to determine the difference in prevalence and characteristics of long COVID symptoms between the two periods and to identify which biological factors were associated with long COVID. |
| Setting | 5 | Describe the setting, locations, and relevant dates, including periods of recruitment, exposure, follow-up, and data collection | 5 | Based on the medical records of the confirmed SARS-CoV-2 RT-qPCR positive cases at Maharat Nakhon Ratchasima Hospital from June to November 2021 (Delta period) and from December 2021 to the first week of August 2022 (Omicron period), all confirmed COVID-19 cases who were ≥ 18 years old, infected by SARS-CoV-2 for the first time within these periods, and had recovered from the infection for a minimum of three months were included as eligible participants. |
| Participants | 6 | (*a*) Give the eligibility criteria, and the sources and methods of selection of participants | 5 | Based on the medical records of the confirmed SARS-CoV-2 RT-qPCR positive cases at Maharat Nakhon Ratchasima Hospital from June to November 2021 (Delta period) and from December 2021 to the first week of August 2022 (Omicron period), all confirmed COVID-19 cases who were ≥ 18 years old, infected by SARS-CoV-2 for the first time within these periods, and had recovered from the infection for a minimum of three months were included as eligible participants. They were approached at random via telephone and requested to provide verbal consent for a single remote interview via telephone. A peer-evaluated questionnaire was employed to record and analyze retrospective self-report data from each participant during the interview process.  Individuals who were pregnant during the COVID-19 infection, missed contact, had no recollection of long COVID experiences, passed away prior to interview, or lacked the ability to communicate in Thai were excluded from the study. |
| Variables | 7 | Clearly define all outcomes, exposures, predictors, potential confounders, and effect modifiers. Give diagnostic criteria, if applicable | 5 | The self-reported questionnaire asked about basic demographic data and the experience of COVID-19 disease, including COVID-19 severity, the presence of long COVID symptoms within three months after infection, drug(s) for treatment, and vaccine status. |
| Data sources/ measurement | 8* | For each variable of interest, give sources of data and details of methods of assessment (measurement). Describe comparability of assessment methods if there is more than one group | 5 | The self-reported questionnaire asked about basic demographic data and the experience of COVID-19 disease, including COVID-19 severity, the presence of long COVID symptoms within three months after infection, drug(s) for treatment, and vaccine status.  A binary logistic regression test with enter method was used to determine the association between biological factors and long COVID outcome. The strength of association was determined using an odds ratio at 95% confidence interval and a P-value < 0.05 was considered as significant association. |
| Bias | 9 | Describe any efforts to address potential sources of bias | 5 | Individuals who were pregnant during the COVID-19 infection, missed contact, had no recollection of long COVID experiences, passed away prior to interview, or lacked the ability to communicate in Thai were excluded from the study. |
| Study size | 10 | Explain how the study size was arrived at | 5 | A minimum sample size of 206 positive COVD-19 cases was required, which was calculated using the n4Studies application (https://www.facebook.com/n4Studies/) based on an estimation infinite population proportion method by setting a prevalence difference proportion of 16% (Ayoubkhani & King, 2022), and an alpha error of 0.05. |
| Quantitative variables | 11 | Explain how quantitative variables were handled in the analyses. If applicable, describe which groupings were chosen and why | 6 | All data were analyzed using the Statistical Package for the Social Sciences (Version 25.0, SPSS Inc., Chicago, IL, USA). Calculation of frequency, percentage, mean and standard deviation was used to characterize the baseline demographic data, to compare the data of long COVID between Delta and Omicron epidemic periods. |
| Statistical methods | 12 | (*a*) Describe all statistical methods, including those used to control for confounding | 6 | All data were analyzed using the Statistical Package for the Social Sciences (Version 25.0, SPSS Inc., Chicago, IL, USA). Calculation of frequency, percentage, mean and standard deviation was used to characterize the baseline demographic data, to compare the data of long COVID between Delta and Omicron epidemic periods, and to compare the genetic variations in the spike protein of the SARS-CoV-2 variants. A binary logistic regression test with enter method was used to determine the association between biological factors and long COVID outcome. The strength of association was determined using an odds ratio at 95% confidence interval and a P-value < 0.05 was considered as significant association. |
|  |  | (*b*) Describe any methods used to examine subgroups and interactions |  |  |
|  |  | (*c*) Explain how missing data were addressed |  |  |
|  |  | (*d*) If applicable, describe analytical methods taking account of sampling strategy |  |  |
|  |  | (*e*) Describe any sensitivity analyses |  |  |
| Results | | |  |  |
| Participants | 13* | (a) Report numbers of individuals at each stage of study—eg numbers potentially eligible, examined for eligibility, confirmed eligible, included in the study, completing follow-up, and analysed | 7 | Demographic characteristics  Between June 2021 and August 2022, during the Delta and Omicron epidemic periods in Thailand, a total of 490 confirmed COVID-19 cases at Maharat Nakhon Ratchasima Hospital were randomly asked for informed consent to participate in a telephone interview. A total of 251 subjects enrolled voluntarily; however, 247 were selected for analysis based on the defined inclusion and exclusion criteria. The number of participants who were infected by SARS-CoV-2 during the Delta and Omicron epidemic periods was 106 and 141, respectively. The flowchart of the subject enrollment process is shown in Figure 1. |
|  |  | (b) Give reasons for non-participation at each stage |  |  |
|  |  | (c) Consider use of a flow diagram |  |  |
| Descriptive data | 14* | (a) Give characteristics of study participants (eg demographic, clinical, social) and information on exposures and potential confounders | 7-9 | The baseline demographics and clinical characteristics of confirmed COVID-19 cases of the Delta and Omicron epidemic periods were compared are shown in Table 1. The mean age of the participants was 40.75 ± 14.20 hears during Delta and 35.61 ± 11.70 years during Omicron. Female gender, age range of 18 to 29 years, obesity, and blood group O were the most prevalent characteristics of the participants in both epidemic periods. Nevertheless, during the Delta period, more than half of the cases (n = 54, or 50.9%) involved immunocompromised hosts who had at least one underlying disease. Hypertension, diabetes, hypercholesterolemia, asthma, and allergies were common underlying conditions. In contrast, infection with SARS-CoV-2 during the Omicron period primarily affected healthy hosts (n = 125, 88.7%). Almost one-third of the Delta cases (n = 38, 35.8%) exhibited symptoms classified as moderate to severe, whereas the Omicron cases predominantly presented mild symptoms. The antiviral drug Favipiravir and symptomatic drug usage was the key treatment utilized by the participants during both variant periods. The majority of the participants affected during the Delta period had not yet been immunized against COVID-19 (n = 63, 59.4%). Conversely, a significant numbers of participants affected during the Omicron period had received at least three vaccinations (n = 80, 56.7%). In respect to the vaccine type, the inactivated vaccine was predominant during the Delta period (n = 24, 55.8%), whereas the mRNA vaccine was predominant during the Omicron period (n = 75, 56.4%). Significantly, throughout both epidemic periods, the majority of participants reported having sustained long COVID, with a number of symptoms having comparable prevalence rates between Delta and Omicron periods.  The prevalence of self-reported long COVID symptoms between the Delta and Omicron epidemic periods was compared (Figure 2). The seven most frequently reported symptoms in long-term COVID patients were as follows: cardiovascular, neurology, dermatology, psychology, respiratory, generalized symptoms of fatigue and myalgia, and others. Both participant groups reported all symptoms but the rate of observation for each symptom varied between the groups. Participants who were infected during the Delta period showed a predominant psychological disorder, including insomnia, anxiety, and depression. Conversely, abnormalities in the respiratory and neuron systems were prevalent during the Omicron period.  A comparison of the baseline demographics of the participants between non-long COVID cases and long COVID cases is presented in Table 2. Non-long COVID cases had a mean age of 40.86 ± 13.74 years and long COVID cases were 36.56 ± 12.59 years old. Comparative frequencies were observed for almost all analyzed variables in the two groups. Significantly, the prevalence of long COVID experience was greater during the Omicron period (60%) compared to the Delta period (40%); additionally, a greater proportion of participants with moderate to severe COVID-19 exhibited a higher rate of long COVID.  A binary logistic regression analysis was used to determine biological factors associated with long COVID. Variant epidemic period, age, treatment with symptomatic medicines, and vaccination status were found to be significant predictors of long COVID outcomes. The odds of developing long COVID were significantly higher for SARS-CoV-2 infection during the Omicron period [OR = 2.976 (95% CI 1.202–7.365); p = 0.018] than for infection during the Delta period. A 1-year increase in age was correlated with a decreased likelihood of developing long COVID [OR = 0.969 (95% CI 0.942–0.996); p = 0.025]. In comparison to other antiviral and immunosuppressive drugs, treatment with symptomatic medications for COVID-19 was associated with a higher risk of developing long COVID [OR = 3.804 (95% CI 1.149–12.590); p = 0.029]. It is worth mentioning that in comparison to vaccination with at least one dose, non-vaccination was a significant risk factor for long COVID [OR = 6.434 (95% CI 1.253–33.033); p = 0.026]. All ten independent variables, as shown in Table 2, possessed the potential to account for 19.3% of the long COVID outcome explanation. The remaining 80.7% of the variance could be accounted for by variables that were not included in the binary logistic regression analysis. The dataset for analysis in this part was available in the supplementary file S1. |
|  |  | (b) Indicate number of participants with missing data for each variable of interest |  |  |
| Outcome data | 15* | Report numbers of outcome events or summary measures |  |  |
| Main results | 16 | (*a*) Give unadjusted estimates and, if applicable, confounder-adjusted estimates and their precision (eg, 95% confidence interval). Make clear which confounders were adjusted for and why they were included |  |  |
|  |  | (*b*) Report category boundaries when continuous variables were categorized |  |  |
|  |  | (*c*) If relevant, consider translating estimates of relative risk into absolute risk for a meaningful time period |  |  |
| Other analyses | 17 | Report other analyses done—eg analyses of subgroups and interactions, and sensitivity analyses | - | Not applicable |
| Discussion | | |  |  |
| Key results | 18 | Summarise key results with reference to study objectives | 11-13 | The potential of a high public health burden due to long COVID must be considered amid the ongoing global transmission of SARS-CoV-2 and its rapidly emerging variants. Research comparing the variations in long COVID prevalence and characteristics caused by distinct virus variants is scarce. Thus, this study collected informative data on long COVID prevalence and clinical symptoms by comparing the Delta and Omicron epidemic periods in Thailand.  Our demographic analysis was consistent with previous studies indicating that most participants in both epidemic periods were female (Antonelli et al., 2022; WHO, 2022). The Omicron variant had a higher potential for infection among healthy people compared to the preceding Delta variant. This may be correlated with the biological characteristic of an increased transmissibility of Omicron. It must be emphasized that all strains of SARS-CoV-2 are capable of inducing long COVID. This is despite the fact that the prevalence of long COVID varied considerably between studies due to dissimilarities in the COVID-19 participant selection process, study sites, ethnicity, self-report bias, and time frame analysis (Antonelli et al., 2022; Du et al., 2022). Over sixty percent of our two participant groups had long COVID. Long COVID prevalence in the Delta period found in this study was comparable with a previous report from Thailand Wangchalabovorn et al. (2022).  Post-acute COVID-19 syndrome or casually long COVID comprises a wide spectrum of clinical symptoms as described in this study. Even though the Delta and Omicron groups presented long COVID characteristics at varying rates, all abnormality classifications were discernible in both groups. As reported in studies from Europe and Asia (Menges et al., 2021; Xiong et al., 2021), fatigue that was categorized as a generalized symptom and respiratory symptoms remained prevalent. Although the precise mechanisms underlying the development of long COVID remain unknown, several hypotheses have been proposed, including immune dysregulation, persistent inflammatory reactions, antibody-dependent enhancement (ADE) from non-neutralizing antibody response, autoimmune mimicry, viral persistence, reactivation of latent pathogens, and alterations in the host microbiome (Batiha et al., 2022; Chen et al., 2023). With respect to viral persistence and shedding, the upper respiratory tract, lower respiratory tract, gastrointestinal tract, and blood were observed to have the longest durations of viral RNA shedding at 83, 59, 126, and 60 days, respectively. (Chen et al., 2023). The sustained presence of SARS-CoV-2 RNA or antigen and immune responses directed against it may determine the development of long COVID (Files et al., 2021; Chen et al., 2023).  In this study, the biological factors that were found significantly associated with the prognosis of long COVID were variant epidemic period, age, treatment with symptomatic medicines, and vaccination status as determined by binary logistic regression analysis. Infection during the Omicron period was associated with an almost three times increased risk of long COVID sequelae in comparison to the Delta period. This finding contradicts the results of previous studies (Antonelli et al., 2022; Du et al., 2022). Although the prevalence of long COVID caused by different strains did not differ significantly, Du et al. (2022) identified a significant distinction in specific symptoms observed in each strain through a systematic review and meta-analysis. As we utilized a lengthy period during the Omicron epidemic covering the variant epidemics of BA.1, BA.2, BA.4, and BA.5, which contain many critical mutations that increased the fitness for infection (Tian et al., 2022), the discrepancy in the period of analysis may account for the outcome of our study. In agreement with the findings of previous studies (Peghin et al., 2021; Maglietta et al., 2022; Notarte et al., 2022; Subramanian et al., 2022; Yoo et al., 2022), advanced age did not support long COVID induction in this analysis. However, it differed from some studies (Sudre et al., 2021; Thompson et al., 2022). Based on our current understanding, this study represents the first report that establishes treatment with symptomatic medications and vaccination status as substantial risk factors for the development of long COVID. As long COVID may be caused by the persistence of viral antigen, which results in an ongoing activation of the host immune response, long COVID can be induced by the use of symptomatic medications lacking antiviral activity. Compared to those who had received at least one dose of vaccination, non-immunized individuals exhibited a six-fold increased risk of developing long COVID. This crucial information was substantiated by a recent cohort research (Catala et al., 2024). Moreover, our analysis data, along with those of other prior studies, emphasized the absence of a correlation between long COVID development and female gender or initial severity of COVID-19 (Townsend et al., 2020; Simani et al., 2021; Al-Kuraishy et al., 2022; Al-Thomali et al., 2022).  **Conclusion**  The SARS-CoV-2 variant, age, treatment with symptomatic medications, and non-immunization status were identified as biological factors associated with long COVID progression in our retrospective cross-sectional study. The identification of these factors should help to facilitate the development of a suitable health management strategy. Furthermore, the analysis of the frequency and biological implications of mutations observed in the spike protein gene of the Delta and Omicron variants could support the explanation for long COVID development and provide a scientific reference for monitoring, prevention, and vaccine development. |
| Limitations | 19 | Discuss limitations of the study, taking into account sources of potential bias or imprecision. Discuss both direction and magnitude of any potential bias |  |  |
| Interpretation | 20 | Give a cautious overall interpretation of results considering objectives, limitations, multiplicity of analyses, results from similar studies, and other relevant evidence |  |  |
| Generalisability | 21 | Discuss the generalisability (external validity) of the study results |  |  |
| Other information | | |  |  |
| Funding | 22 | Give the source of funding and the role of the funders for the present study and, if applicable, for the original study on which the present article is based | - | This research received no specific grant from any funding agency in the public, commercial, or not-for-profit sectors. |

*Give information separately for exposed and unexposed groups.

**Note:** An Explanation and Elaboration article discusses each checklist item and gives methodological background and published examples of transparent reporting. The STROBE checklist is best used in conjunction with this article (freely available on the Web sites of PLoS Medicine at http://www.plosmedicine.org/, Annals of Internal Medicine at http://www.annals.org/, and Epidemiology at http://www.epidem.com/). Information on the STROBE Initiative is available at www.strobe-statement.org.
